# Hygroscopic Coating of Sulfuric Acid Shields Oxidant Attack on the Atmospheric Pollutant Benzo(a)pyrene Bound to Model Soot Particles

**DOI:** 10.1038/s41598-017-18292-z

**Published:** 2018-01-09

**Authors:** Debajyoti Ray, Tara Shankar Bhattacharya, Abhijit Chatterjee, Achintya Singha, Sanjay K. Ghosh, Sibaji Raha

**Affiliations:** 1 Environmental Sciences Section, Bose Institute, P 1/12 CIT Scheme VII-M, Kolkata, 700054 India; 2 Department of Physics, Bose Institute, 93/1, A.P.C Road, Kolkata, 700009 India; 3 Centre for Astroparticle Physics and Space Science, Block-EN, Sector-V, Salt Lake, Kolkata, 700091 India

## Abstract

Substantial impacts on climate have been documented for soot‒sulfuric acid (H_2_SO_4_) interactions in terms of optical and hygroscopic properties of soot aerosols. However, the influence of H_2_SO_4_ on heterogeneous chemistry on soot remains unexplored. Additionally, oxidation rate coefficients for polycyclic aromatic hydrocarbons intrinsic to the atmospheric particles evaluated in laboratory experiments seem to overestimate their degradation in ambient atmosphere, possibly due to matrix effects which are hitherto not mimicked in laboratory experiments. For the first time, our kinetics study reports significant influence of H_2_SO_4_ coating on heterogeneous ozonation of benzo(a)pyrene (BaP) deposited on model soot, representative to atmospheric particles. The approximate specific surface area of model soot (5 m^2^g^−1^) was estimated as a measure of the availability of surface molecules to a typical gaseous atmospheric oxidant. Heterogeneous bimolecular reaction kinetics and Raman spectroscopy studies suggested plausible reasons for decreased BaP ozonation rate in presence of H_2_SO_4_: 1. decreased partitioning of O_3_ on soot surface and 2. shielding of BaP molecules to gaseous O_3_ by acid-BaP reaction or O_3_ oxidation products.

## Introduction

Synchronous to the rapid pace of urbanization and amplified energy demand, atmospheric pollution with organic toxicants such as polycyclic aromatic hydrocarbons (PAHs) intrinsic to carbonaceous particles or soot, is rapidly enhancing^1^. Emission of soot^2^ and PAHs occur from diverse sources which are mainly anthropogenic, for example, incomplete combustion of fossil fuels and biomass in transport, residential, agriculture and commercial sectors. Soot particles absorb and scatter solar radiation influencing the earth’s radiative budget^3,4^ and the co-emitted organic toxicants pose severe threats to human health^5^. In fact, laboratory studies have shown that toxicity of the soot surface composition was enhanced by 1.5–2 times upon heterogeneous ozone oxidation^6^. Freshly emitted soot aggregates are composed of hydrophobic spherules which undergo aging by adsorption or condensation of either directly H_2_SO_4_ vapor or gaseous SO_2_ which eventually ends at H_2_SO_4_ and leads to a highly hygroscopic coating on the soot particles^7^. However, Donaldson and coworkers recently argued for the formation of hygroscopic sulfurous acid (H_2_SO_3_) from triplet SO_2_ as it is difficult for ground state SO_2_ to cross the high activation barrier of the endothermic reaction^8^. Nevertheless, the soot particles with hygroscopic coating might act as an effective nucleus to form small water clusters and subsequently grow in size by rapid condensation of more water molecules^9^. Indeed, H_2_SO_4_ coating on propane soot particles enhanced their hygroscopic size and the particles could act as cloud condensation nuclei (CCN) at 80% relative humidity condition^7^. Additionally, heterogeneous oxidation by hydroxyl radicals, ozone and nitrogen oxides can also aid to enhanced hydrophilicity of the aerosol surface by increasing oxygen- and nitrogen-containing functional groups in the surface adsorbed organic species^10^. Despite considerable studies on atmospheric aging of soot and subsequent alteration of their physicochemical properties such as morphology, hygroscopicity and optical properties, kinetic studies on heterogeneous oxidation of organics on soot surface are limited and the subject is still poorly understood^11^. Notably, the studies of Poschl and coworkers reported a non-linear Langmuir type dependence of benzo(a)pyrene (BaP, a 5-ring PAH; PAHs are ubiquitous organic pollutants) ozonation rate with gaseous ozone^12^. Later, studies involving 2–5 ring PAHs associated with wide range of substrates including solid carboxylic acid aerosols^13^, dry sodium chloride aerosols^13^, ammonium sulfate particles^14^, pyrex glass^15^, thin film of carboxylic acids^16^, water^17^ and 1-octanol^17^ reported substrate specific but similar non-linear relationships between ozonation rate constant and gaseous ozone concentration, which is consistent with the Langmuir-Hinshelwood model involving an initial rapid equilibration of gaseous ozone on substrate surface, followed by heterogeneous oxidation of PAHs^13^. So far laboratory studies are focused on the ozonation of PAHs adsorbed on single component surface but in the real-world scenario, the surface composition of atmospheric particles is complex, consisting of both organic and inorganic components.

The current work is the experimental manifestation of a hitherto assumption that shielding effect extends pollutants’ lifetime in the real environment. From the mechanistic consideration of the Langmuir-Hinshelwood kinetic model, we investigated whether a hygroscopic coating of H_2_SO_4_ can influence the ozonation kinetics of BaP deposited on laboratory generated soot particles. The approximate specific surface area of the soot particles was estimated not by the accessibility of soot surface but by the availability of surface-bound BaP molecules to gaseous ozone. In addition, the soot samples were characterized by Raman spectroscopy.

## Results

To increase reproducibility of the experiments, the organic and inorganic impurities from the collected soot particles generated in controlled combustion of kerosene were removed chemically and thermally (see Supplementary information, pageS3). The resulting soot particles were termed as *cleaned soot*. The cleaned soot particles coated with BaP were termed as soot_BaP_ and the soot_BaP_ particles coated with H_2_SO_4_were termed as ${{\rm{soot}}}_{{\rm{BaP}}+{{\rm{H}}}_{2}{{\rm{SO}}}_{4}}$.

### Sample characterization by Raman Spectroscopy

**Figure 1:**
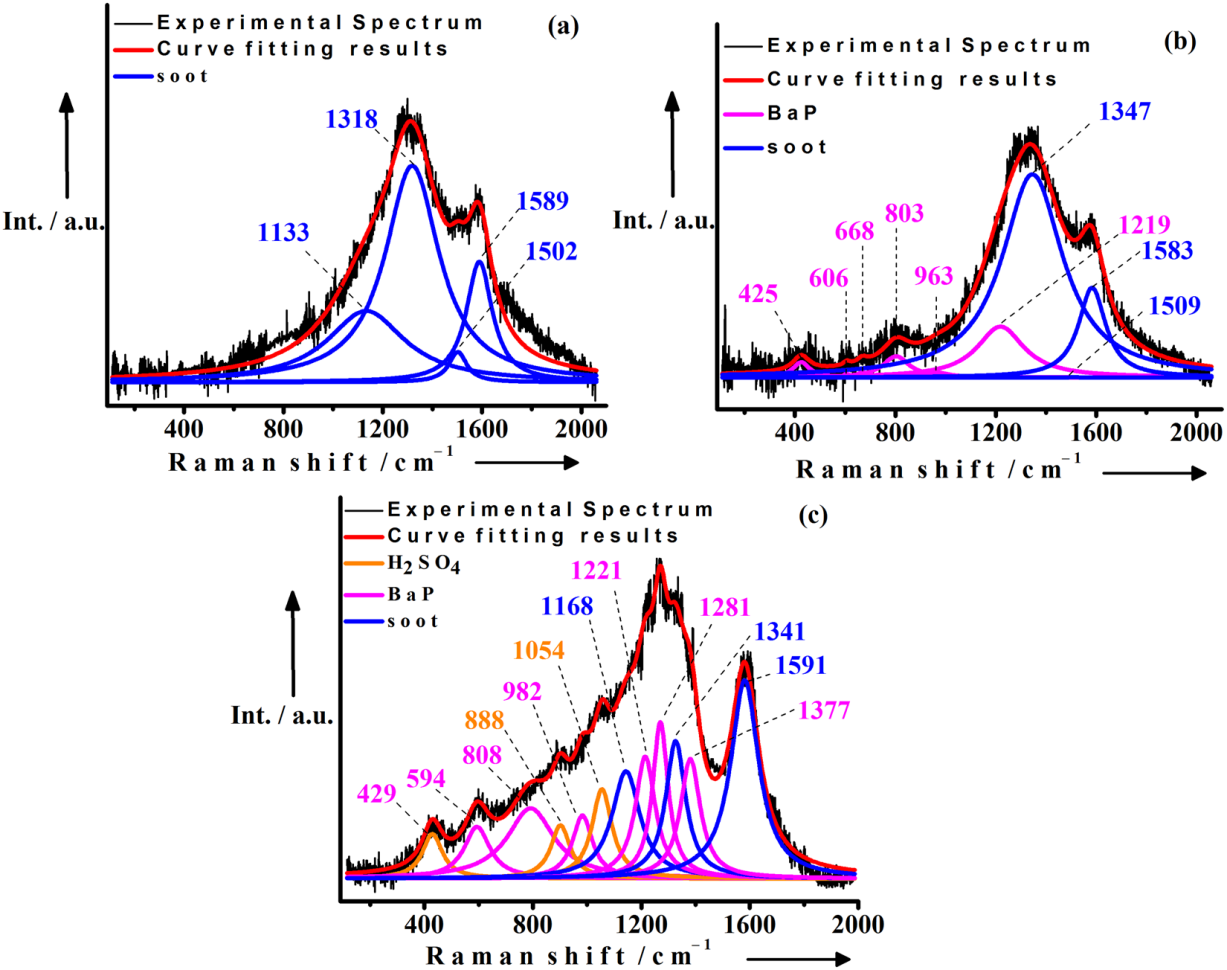
Characterization of soot samples prior to heterogeneous ozonation reaction. Curve fitting results for the typical first order Raman spectra of (1a) cleaned soot (based on four bands); (1b) cleaned soot coated with benzo(a)pyrene (BaP) (based on nine bands); (1c) cleaned soot first coated with BaP followed by coated with H_2_SO_4_ (based on twelve bands). The peak positions of soot, BaP and H_2_SO_4_ are indicated in the graphs.

In order to have a better understanding of the surface properties of the cleaned soot, soot_BaP_ and ${{\rm{soot}}}_{{\rm{BaP}}+{{\rm{H}}}_{2}{{\rm{SO}}}_{4}}$ samples at the molecular level, we have performed Raman measurements. Figure 1(a‒c) provide the Raman spectra of cleaned soot, soot_BaP_ and ${{\rm{soot}}}_{{\rm{BaP}}+{{\rm{H}}}_{2}{{\rm{SO}}}_{4}}$ respectively. The Raman spectrum of cleaned soot (Fig. 1a) is well fitted with four Lorentzian shaped bands centered at 1133, 1318, 1502 and 1589 cm^−1^. The bands are generated due to different structural and compositional types in the soot sample^18,19^. The mode at ~1589 cm^−1^ (G-band) is the graphitic lattice vibration with E_2g_ symmetry. The band at ~1318 cm^−1^ corresponds to disordered graphite lattice with A_1g_ symmetry mode. The observed band at ~1502 cm^−1^ represents the amorphous fraction of the soot sample. The peak at ~1133 cm^−1^ corresponds to C–C and C=C bond stretching vibration of polyene like structure with A_1g_ symmetry. Nine bands are identified in the deconvoluted Raman spectra of soot_BaP_ (Fig. 1b) prior to the exposure to gaseous ozone. Apart from the peaks due to carbonaceous soot (vide supra), we observed additional six modes at 425 cm^−1^, 606 cm^−1^, 668 cm^−1^, 803 cm^−1^, 963 cm^−1^ and 1219 cm^−1^, which are attributed to the different types of vibrations of BaP molecules^20^. The origins of these vibrational bands are summarized in Table 1. Prior to the exposure to gaseous ozone, twelve bands are identified in the deconvoluted Raman spectra of ${{\rm{soot}}}_{{\rm{BaP}}+{{\rm{H}}}_{2}{{\rm{SO}}}_{4}}$(Fig. 1c); details of the band assignments are given in Table  1. Again, apart from the peaks due to carbonaceous soot and BaP, the modes at 888 cm^−1^ and 1054 cm^−1^ are identified as the vibrational bands of H_2_SO_4_^21^.

**Table 1:** Assignment of vibrational bands of the Raman spectra of cleaned soot, soot_BaP_ and ${\rm{s}}{\rm{o}}{\rm{o}}{{\rm{t}}}_{{\rm{B}}{\rm{a}}{\rm{P}}+{{\rm{H}}}_{2}{\rm{S}}{{\rm{O}}}_{{\rm{4}}}}$.

Sample	Wave number (cm^−1^)	Assigned vibrational mode	References
cleaned soot	1133	C─C and C ═ C bond stretching vibration of polyene like structure with A_1g_ symmetry	^18^^,^^19^
	1318	disordered graphite lattice with A_1g_ symmetry	
	1502	amorphous fraction of the soot sample	
	1589	ideal graphitic lattice vibration mode with E_2g_ symmetry (G band)	
soot_BaP_	425	out of plane ring bending vibrations of BaP	^18^^,^^19,^ ^20^^,^^42^
	606, 668, 803, 963	CH out of plane bending of BaP	
	1219	CH bending of BaP	
	1347	disordered graphite lattice with A_1g_ symmetry mode	
	1509	amorphous fraction of the soot sample	
	1583	ideal graphitic lattice vibration mode with E_2g_ symmetry (G band)	
${{\rm{soot}}}_{{\rm{BaP}}+{{\rm{H}}}_{2}{{\rm{SO}}}_{4}}$	429	out of plane ring bending vibrations of BaP	^18–21,41,42^
	594	CH out of plane bending of BaP	
	808	CH out of plane bending of BaP	
	888	asymmetric stretching vibration of ${{\rm{HSO}}}_{4}^{-}$	
	982	CH out of plane bending of BaP	
	1054	symmetric stretching vibration of ${{\rm{HSO}}}_{4}^{-}$ ions	
	1168	C─C and C ═ C bond stretching vibration of polyene like structure with A_1g_ symmetry	
	1221,1281	CH bending of BaP	
	1341	disordered graphite lattice with A_1g_ symmetry mode	
	1377	strong CH in-plane bending coupled with weak ring breathing of BaP	
	1591	ideal graphitic lattice vibration mode with E_2g_ symmetry (G band)	

### Determination of specific surface area of soot

Surface coverage is the fraction of total number of surface active sites of an adsorbent occupied by adsorbate molecules. Previous studies have shown that substrate surface coverage significantly influence the reaction kinetics. For example, Alebic-Juretic and coworkers showed ozonation rate enhancement by a factor of 2.36 at sub-monolayer surface coverage of BaP on non-activated silica gel^22^. Similar observations have been reported for other substrates, such as spark discharge soot particles^12^ and azelaic acid aerosols^13^. Therefore, the monolayer surface coverage of BaP on the soot_BaP_ samples was determined by using the technique utilized by Ray and coworkers for artificial snow samples^23^. Analogous to those authors, the Langmuir concentration (${c}_{{\rm{BaP}}}^{{\rm{L}}}$), i.e, the concentration corresponding to the monolayer coverage of BaP on soot surface was evaluated.

**Figure 2:**
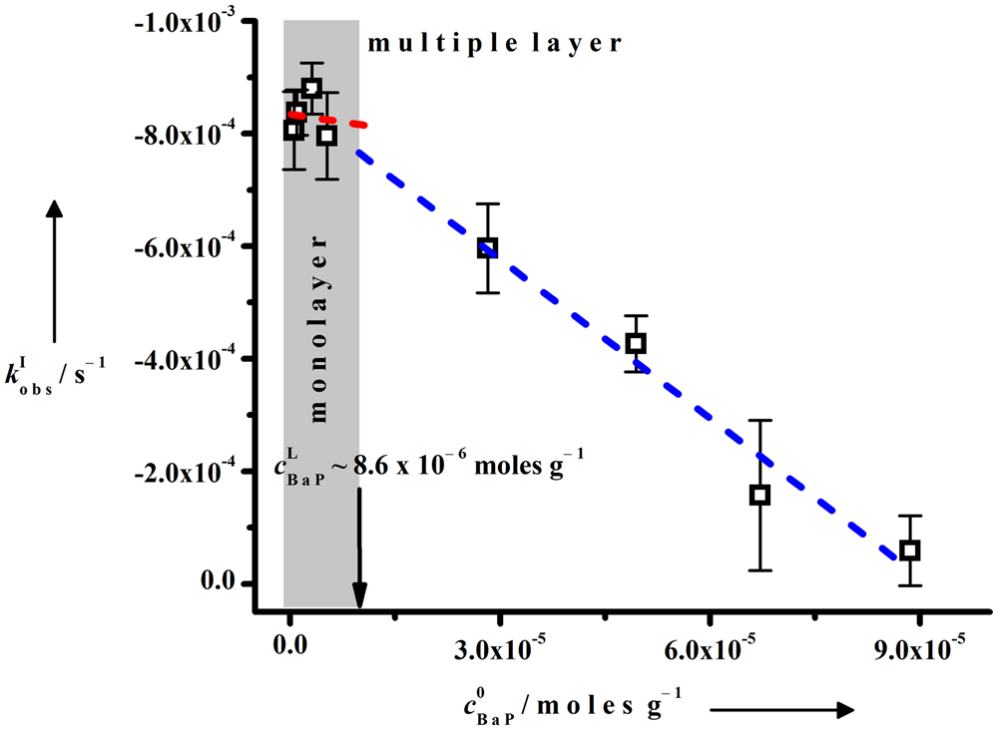
Determination of Langmuir concentration from kinetic experimental data. BaP ozonation kinetics on soot_BaP_ samples as a function of ${c}_{{\rm{B}}{\rm{a}}{\rm{P}}}^{0}$ inside a quartz glass reactor at room temperature and pressure; ${c}_{{{\rm{O}}}_{3}}$= 2 × 10^14^ molecules cm^−3^; the evaluated ${k}_{{\rm{obs}}}^{{\rm{I}}}\,$values were corrected for losses due to evaporation and O_2_ oxidation; error bars represent the standard deviation.

The BaP ozontion kinetics was studied by quantifying the BaP degradation in soot_BaP_ samples with a series of BaP surface loads at ~8 ppm ozone concentration with increasing ozone exposure time (Fig. S2). Figure 2 shows the plot of observed pseudo-first order rate constants, ${k}_{{\rm{obs}}}^{{\rm{I}}}$ values (from Supplementary Fig. S2) against the corresponding BaP surface loads. The ${k}_{{\rm{obs}}}^{{\rm{I}}}$ values are independent at lower BaP surface loads (sub-monolayer), whereas decreasing ${k}_{{\rm{obs}}}^{{\rm{I}}}$ values are observed at higher BaP surface loads (above monolayer). The Langmuir concentration ${c}_{{\rm{BaP}}}^{{\rm{L}}}$ = $8.6\,\times \,{10}^{-6}{\text{moles}g}^{-1}$ was estimated from the intersection point of two linear least-squares-fits with clearly different slopes^22,24^.

Alebic-Juretic and coworkers explained their observation of ozonation rate enhancement at sub-monolayer BaP surface load by postulating rapid desorption of BaP oxidation products from the surface and chemical activation of BaP by the acidic silica gel surface^22^. On the contrary, Poschl and coworkers argued that most of the possible BaP oxidation products are not volatile enough for rapid desorption from silica gel surface^12^. Furthermore, these authors suggested that instead of rate enhancement at sub-monolayer BaP coverage, the reaction rates were reduced at above-monolayer surface loads due to burial effects by multiple layers of BaP and its oxidation products. Indeed, the gaseous ozone molecules are unable to reach the underlying BaP molecules at above monolayer surface load of BaP whereas at sub-monolayer condition the ${k}_{{\rm{obs}}}^{{\rm{I}}}$ values remain independent of the BaP surface loads, resulting in two distinct slopes in Fig. 2.

Additionally, the ${c}_{{\rm{BaP}}}^{{\rm{L}}}$ value was utilized to calculate the specific surface area (SSA) which is the gas accessible area of unit mass of a solid^23^. Since the accessibility of surface adsorbed BaP molecules to the gaseous ozone was considered instead of the soot surface, the SSA evaluated in this work represents a relative value. Therefore we termed it *approximate specific surface area* (ASSA). The ASSA of the soot particles was estimated to be 5 m^2^g^−1^, using equation (1) ^23,24^:

1 $${\rm{A}}{\rm{S}}{\rm{S}}{\rm{A}}={c}_{{\rm{B}}{\rm{a}}{\rm{P}}}^{{\rm{L}}}\times {{\rm{N}}}_{{\rm{A}}}\times {{\rm{A}}}_{{\rm{B}}{\rm{a}}{\rm{P}}}$$

where, ${c}_{{\rm{BaP}}}^{{\rm{L}}}$ is the Langmuir concentration, N_A_ is Avogadro’s number and A_BaP_ is the molecular cross-sectional area of BaP^15^ assumed to be 1 nm^2^. Numerous studies have measured the specific surface area (SSA) of soot from various sources mainly by using the standard Brunauer–Emmett–Teller (BET) model for N_2_ adsorption isotherm, where N_2_ molecules undergo physical adsorption on soot surface^25^. In fact, the SSA determined by the BET technique is a measure of the gas accessible surface area of a solid. The SSA of soot particles generated from a wide range of sources ranged between 0.1–500 m^2^ g^−1^ ^26^. Although the magnitude of ASSA of our model soot (~5 m^2^ g^−1^) is found small compared to spark discharge soot (395 m^2^ g^−1^)^12,26^, however, comparable values are observed for other types of soot which are more representative of the real-world atmosphere, such as wood stove^27^ (1.0 m^2^ g^−1^), bus exhaust^27^ (1.9 m^2^ g^−1^), marine vessel exhaust^27^ (12 m^2^ g^−1^) and soot of black smoke from ceramic furnace flue gas^28^ (15 m^2^ g^−1^). Also BET-Kr adsorption isotherm has been used to estimate the SSA of aviation kerosene soot (43 m^2^ g^−1^)^29^.

### Heterogeneous ozonation kinetics of BaP in soot_BaP_ and ${\bf{s}}{\bf{o}}{\bf{o}}{{\bf{t}}}_{{\bf{B}}{\bf{a}}{\bf{P}}+{{\bf{H}}}_{2}{\bf{S}}{{\bf{O}}}_{4}}$ samples

**Figure 3:**
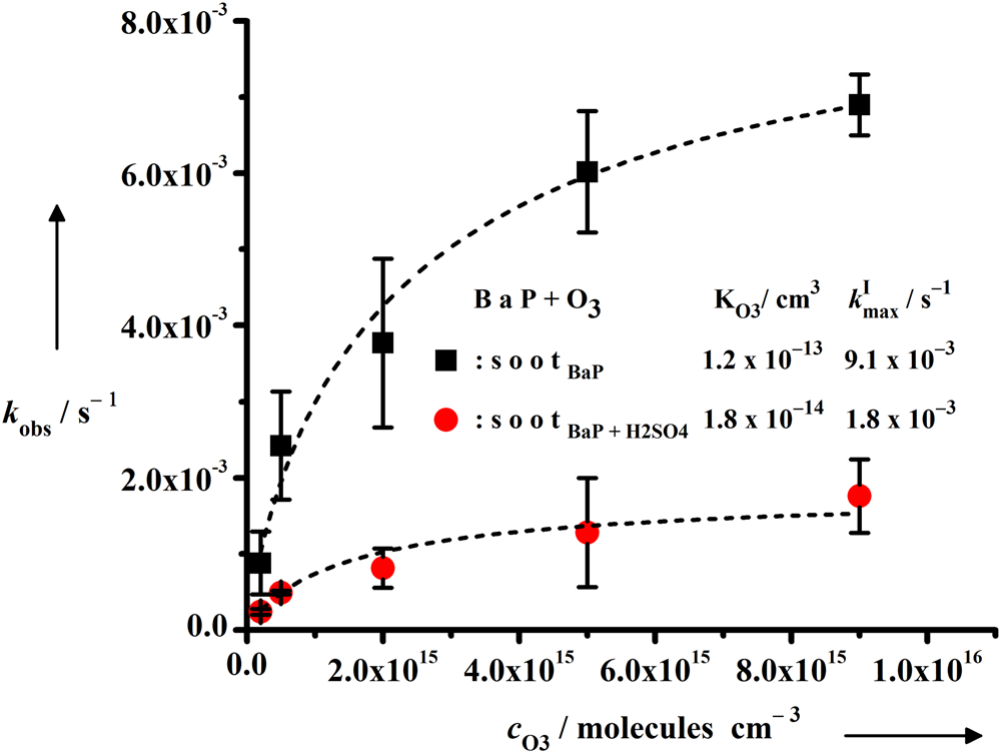
Bimolecular heterogeneous reaction on soot surface. Pseudo first-order rate constants (${k}_{{\rm{obs}}}^{{\rm{I}}}$) as a function of gaseous ozone concentration for the reaction of BaP (${c}_{{\rm{Bap}}}^{0}$ = $3.12\,\times \,{10}^{-6}{\text{moles}g}^{-1}$) and ozone on clean and H_2_SO_4_ coated soot inside the reactor at room temperature and pressure; error bars represent the standard deviation. The ${{\rm{K}}}_{{{\rm{O}}}_{3}}\,$and *k*_max_values were evaluated by fitting the plots with Langmuir ─ Hinshelwood equation.

The ozonation kinetics of sub-monolayer concentration of BaP (${c}_{{\rm{B}}{\rm{a}}{\rm{P}}}^{0}$ = $3.12\,\times \,{10}^{-6}{\text{moles}g}^{-1}$, corresponding to ~0.4 times monolayer) adsorbed on soot_BaP_ and ${{\rm{soot}}}_{{\rm{BaP}}+{{\rm{H}}}_{2}{{\rm{SO}}}_{4}}$ samples, were studied by exposing to a range of ozone concentrations, ${c}_{{{\rm{O}}}_{3}}$=8─360 ppm (see Supplementary Fig. S3). The evaluated ${k}_{{\rm{obs}}}^{{\rm{I}}}$ values from Fig. S3a and b were then plotted against the corresponding ozone concentrations in Fig. 3. The non-linear relationship between ${k}_{{\rm{obs}}}^{{\rm{I}}}$ and ${c}_{{{\rm{O}}}_{3}}$ indicates heterogeneous reaction on both soot_BaP_ and ${{\rm{soot}}}_{{\rm{BaP}}+{{\rm{H}}}_{2}{{\rm{SO}}}_{4}}$ samples. Figure 3 also demonstrates that initially ${k}_{{\rm{obs}}}^{{\rm{I}}}$ values are increasing linearly with increasing ${c}_{{{\rm{O}}}_{3}}$ but become independent and leveled off at higher ${c}_{{{\rm{O}}}_{3}}$ implying the Langmuir-Hinshelwood (LH) type of kinetics. The LH model^30^ demonstrates heterogeneous bimolecular reaction involving rapid initial equilibrium partitioning of O_3_ at the air-soot interface prior to heterogeneous reaction between BaP and O_3_^31^. The experimental results of Shiraiwa and coworkers demonstrated possible involvement of long-lived reactive oxygen intermediates in the LH mechanism^32^. Therefore the plots in Fig. 3 were fitted with modified LH equation, equation (2)^15^:

2 $${k}_{{\rm{obs}}}^{{\rm{I}}}={k}_{{\rm{\max }}}\frac{{{\rm{K}}}_{{{\rm{O}}}_{3}}[{{\rm{O}}}_{3}]}{1+{{\rm{K}}}_{{{\rm{O}}}_{3}}[{{\rm{O}}}_{3}]}$$

where, *k*_max_ is the maximum pseudo-first order rate constant at saturated surface concentration of ozone, ${{\rm{K}}}_{{{\rm{O}}}_{3}}$ is the adsorption equilibrium constant of ozone, and [O_3_] is the gas phase ozone concentration. The partitioning of gaseous ozone was observed to be higher by more than 1 order of magnitude on azelaic acid aerosol (${{\rm{K}}}_{{{\rm{O}}}_{3}}$ = ~ $1.2\,\times \,{10}^{-15}$ cm^3^) and by 3 orders of magnitudes on spark discharge soot (${{\rm{K}}}_{{{\rm{O}}}_{3}}$= $2.8\,\times {10}^{-13}$ cm^3^)^12^ than that on NaCl surface (${{\rm{K}}}_{{{\rm{O}}}_{3}}$< $1.2\,\times {10}^{-16}$ cm^3^)^13^, implying that ozone has higher affinity for non-polar surfaces. In good agreement with the previous results, we observed that ozone partitioning was ~ 7 times higher on non-polar soot_BaP_ surface (${{\rm{K}}}_{{{\rm{O}}}_{3}}$ = $1.2\,\times \,{10}^{-13}$ cm^3^) than that on relatively polar surface of the ${{\rm{soot}}}_{{\rm{BaP}}+{{\rm{H}}}_{2}{{\rm{SO}}}_{4}}\,$samples (${{\rm{K}}}_{{{\rm{O}}}_{3}}$ = $1.8\times {10}^{-14}$ cm^3^) due to presence of H_2_SO_4_. Thus our results support Zhang and coworkers who concluded that uptake of H_2_SO_4_ on model soot surface, converted the hydrophobic soot into hydrophilic aerosol^33^. Also the evaluated ${{\rm{K}}}_{{{\rm{O}}}_{3}}\,$value of our model soot varied within a factor of 2.5 from the ${{\rm{K}}}_{{{\rm{O}}}_{3}}$ value evaluated by Poschl and coworkers^12^. Previous studies have shown that ${{\rm{K}}}_{{{\rm{O}}}_{3}}$ may vary by 3 orders of magnitude but the typical range of *k*_max_ falls within (${10}^{-3}-{10}^{-2}){{\rm{s}}}^{-1}$ and varies by a factor of 3. The *k*_max_ for soot_BaP_(*k*_max_ = $9.12\,\times {10}^{-3}{{\rm{s}}}^{-1}$) and ${{\rm{soot}}}_{{\rm{BaP}}+{{\rm{H}}}_{2}{{\rm{SO}}}_{4}}$ (*k*_max_ = $1.85\times {10}^{-3}{{\rm{s}}}^{-1}\,)$ samples are within this range. This is indicative of a similar rate-determining step for BaP ozonation on model soot surface, possibly involving formation of reactive oxygen intermediates from ozone, as postulated by Shiraiwa and co-workers^32^. The *k*_max_ for soot_BaP_ samples was found to be ~5 times higher than ${{\rm{soot}}}_{{\rm{BaP}}+{{\rm{H}}}_{2}{{\rm{SO}}}_{4}}\,$samples. Indeed, the possibility of slow bulk reaction on ${{\rm{soot}}}_{{\rm{BaP}}+{{\rm{H}}}_{2}{{\rm{SO}}}_{4}}$ samples is most unlikely because the nonlinear shape of the plot of ${k}_{{\rm{obs}}}^{{\rm{I}}}$ as a function of ${c}_{{{\rm{O}}}_{3}}$ (Fig. 3) is consistent with LH mechanism indicative of a heterogeneous bimolecular reaction^13^. According to LH model, limited numbers of active sites are available for air-soot surface partitioning of gaseous ozone. A saturated condition is developed at some ozone concentration when all the active surface sites are occupied. Beyond this saturation point, the ozonation rate constant should become independent of the ozone concentration. This is clearly illustrated in Fig. 3, where the *k*_max_ forms a plateau at high ozone concentrations.

Mmereki and co-workers studied anthracene (3-ring PAH) ozonation kinetics and observed that in presence of near-monolayer coating of a series of *n*-carboxylic acid films, the *k*_max_ of the reaction between gaseous ozone and surface adsorbed anthracene on air-aqueous interface decreased significantly in comparison to uncoated water surface^16^. The authors postulated the formation of organic acid and anthracene complex at the ozone attacking positions of anthracene molecules. Consequently both ozone and organic acids are in competition for the same reaction site resulting in reduction of ozonation rate. This reasoning can be assumed to be partly effective to explain our observations. If H_2_SO_4_─BaP complex formation were the only reason for slower ozonation rate in our study then we would not have observed >70% BaP recovery in prolonged ozone exposure of ${{\rm{soot}}}_{{\rm{BaP}}+{{\rm{H}}}_{2}{{\rm{SO}}}_{4}}$ samples (Supplementary Fig. S4). Moreover Henning and coworkers reported that H_2_SO_4_ possibly consumes PAHs to produce lower molecular weight products^34^. Additionally, the degree of disorder in soot which is measured from the intensity ratio of defect (D) and ideal graphite (G) bands^35^, i.e., I_D_/I_G_in cleaned soot, soot_BaP_ and ${{\rm{soot}}}_{{\rm{BaP}}+{{\rm{H}}}_{2}{{\rm{SO}}}_{4}}$ are estimated to be 1.79, 4.8 and 0.51 respectively. The I_D_/I_G_ ratio also corresponds to the aromatic/olefinic ration of a sample^36,37^. Thus relative to the I_D_/I_G_ value of cleaned soot (1.79), the higher I_D_/I_G_ value for soot_BaP_ (4.8) clearly indicates the presence of BaP on the soot surface, as D bands arise from larger aromatic compounds^38^. Interestingly, I_D_/I_G_ ratio is reduced significantly when H_2_SO_4_ was coated on soot_BaP_. Indeed, our Raman spectroscopic measurements (Fig. 1) shows that the I_D_/I_G_ value i.e., aromatic/olefinic ratio is significantly reduced for ${{\rm{soot}}}_{{\rm{BaP}}+{{\rm{H}}}_{2}{{\rm{SO}}}_{4}}$(I_D_/I_G_ = 0.51) when the soot_BaP_ (I_D_/I_G_ = 4.8) samples were coated with H_2_SO_4_. Therefore possibly the H_2_SO_4_–BaP complex and/or products formed from the reaction between H_2_SO_4_ and BaP in ${{\rm{soot}}}_{{\rm{BaP}}+{{\rm{H}}}_{2}{{\rm{SO}}}_{4}}$ samples, hinder the BaP molecules to gaseous ozone resulting in the slow reaction rate compared to that in soot_BaP_ samples. Essentially, extensive investigations are required further to decipher the reasons of slowing down of BaP ozonation rate in presence of H_2_SO_4_, inconspicuously.

In this experimental study we have focused on a hitherto unexplored topic of whether the hygroscopic coating on soot aerosol surface influences the heterogeneous oxidation kinetics and explored its consequence. We have shown in this paper for the first time that H_2_SO_4_ coating markedly influences the oxidation kinetics of soot surface adsorbed BaP by gaseous ozone. The maximum first order rate constants (*k*_max_) of BaP ozonation reaction on soot_BaP_ and ${{\rm{soot}}}_{{\rm{BaP}}+{{\rm{H}}}_{2}{{\rm{SO}}}_{4}}\,$samples were found to be $9.12\,\times {10}^{-3}{{\rm{s}}}^{-1}$ and $1.85\,\times \,{10}^{-3}{{\rm{s}}}^{-1}$ respectively. Thus *k*_max_ was reduced by nearly 5 times in presence of H_2_SO_4_ coating. In immediate effect, the half-life of soot surface adsorbed BaP is enhanced, as calculated for soot_BaP_ (*t*_1/2_ ~17 minutes) and ${{\rm{soot}}}_{{\rm{BaP}}+{{\rm{H}}}_{2}{{\rm{SO}}}_{4}}$(*t*_1/2_ ~2 hours) samples at atmospherically relevant ozone concentration of 100 ppb^15^. If this is a general phenomenon then the lifetime enhancement would possibly result in long range transport of soot bound chemicals causing pollution to a pristine area^14^. On the other hand, particles in ambient air affect the atmosphere directly by scattering or absorbing solar radiation as well as indirectly by forming cloud condensation nuclei (CCN), affecting the microphysical properties of cloud^4,7^. Therefore, at ambient conditions the aged soot particles can also act as CCN and be subsequently removed by rain, resulting in soil or water pollution^39^. Ageing of soot particles can also bring changes in cloud albedo (Twomey effect) and precipitation pattern^34^. It is believed that the experimental rate coefficient values are possibly overestimations compared to the real atmospheric values^40^, because in real atmosphere, particle associated PAHs are shielded from atmospheric oxidants which have not yet been mimicked in the experiments. BaP apparently undergoes faster chemical degradation compared to that in real atmosphere. The H_2_SO_4_ coating in the ${{\rm{soot}}}_{{\rm{BaP}}+{{\rm{H}}}_{2}{{\rm{SO}}}_{4}}\,$samples however, reflects a multiphase condition to the adsorbed BaP molecules and thus our study is undoubtedly a successful attempt in approaching the real atmospheric conditions. Furthermore, development of a simple soot preparation method from kerosene and subsequent characterization of the soot samples by Raman spectroscopy and validation by evaluating their approximate specific surface area (~5 m^2^g^−1^) followed by utilization of the soot samples for kinetics experiments are also novel aspects of this study. Further environmental implications of this work are currently being characterized using models and will be reported in due course.

## Methods

Soot particles were generated under controlled combustion of kerosene in a set up similar to that of a typical wick lamp (Fig. S1a). Length of the wick was adjusted to maintain a stable and medium flame. Ultra high purity air (flow rate was adjusted at 0.3 ${{\rm{L}}\min }^{-1}$ for maximum soot collection), carrying the soot particles, was bubbled through 200 mL n-hexane, where the particles were deposited. The collected particles were further washed by dichloromethane (DCM) followed by heated for 5 hrs inside a furnace at 400 °C to ensure maximum removal of the organic impurities. These *cleaned soot* particles were used as the substrate for heterogeneous ozonation of BaP. The soot_BaP_ samples were prepared by soaking the *cleaned soot* into different concentrations of BaP in DCM, followed by blowing off DCM under gentle stream of nitrogen gas. The ${{\rm{soot}}}_{{\rm{BaP}}+{{\rm{H}}}_{2}{{\rm{SO}}}_{4}}$ samples were prepared by soaking 10 g soot_BaP_ particles into 10 mL ~1 μM H_2_SO_4_ solution followed by drying off the particles by heating at 180 °C. An experimental set up was developed to study the heterogeneous BaP ozonation kinetics (see Supplementary Fig. S1). In brief, ozone was generated by flowing ultra-high purity grade O_2_ gas through non-thermal plasma generated inside a homemade dielectric barrier discharge (DBD) reactor. The soot_BaP_ and ${{\rm{soot}}}_{{\rm{BaP}}+{{\rm{H}}}_{2}{{\rm{SO}}}_{4}}\,$samples were simultaneously exposed to gaseous O_3_ inside a quartz glass tube reactor. The ozonation kinetics was determined by evaluating the BaP degradation with O_3_ exposure time. The initial and unreacted BaP concentrations were measured using high performance liquid chromatography (HPLC, Shimadzu Prominance), equipped with a C-18 reversed-phase chromatographic column (SupelcosilTM LC-PAH, 15 cm × 4.6 mm, 5 µm) and a fluorescence detector (Shimadzu RF 10AXL). Raman measurements were performed in backscattering geometry using LabRAM HR (Jobin Yvon) spectrometer equipped with a Peltier-cooled charge-coupled-device (CCD) detector. An air cooled argon ion laser with a wavelength of 488 nm was used as the excitation light source. Raman spectra of all samples have been recorded in the frequency range of 50–2000 cm^−1^ under similar experimental conditions.

## Electronic supplementary material

Supplementary Information
