# Supplementary material for: Hygroscopic Coating of Sulfuric Acid Shields Oxidant Attack on the Atmospheric Pollutant Benzo(a)pyrene Bound to Model Soot Particles
Source: Sci Rep. 2018 Jan 9;8:129. doi: 10.1038/s41598-017-18292-z (PMC5760694; doi:10.1038/s41598-017-18292-z)
Supplement: Supplementary file 1 — Supplementary Information [file 41598_2017_18292_MOESM1_ESM.pdf]

Supplementary Information

Hygroscopic Coating of Sulfuric Acid Shields Oxidant Attack on the Atmospheric Pollutant Benzo(a)pyreneBound to Model Soot Particles

Debajyoti Ray[1], Tarashankar Bhattacharya[2], Abhijit Chatterjee[1,3], Achintya Singha[2], Sanjay K. Ghosh[2,3] & Sibaji Raha*[1,2,3]

[1]Environmental Sciences Section, Bose Institute, P 1/12 CIT Scheme VII-M, Kolkata-700054, India, [2]Department of Physics, Bose Institute, 93/1, A.P.C Road, Kolkata-700009, India, [3]Centre for Astroparticle Physics and Space Science, Block-EN, Sector-V, Salt Lake, Kolkata-700091, India

e-mail: sibaji.raha@jcbose.ac.in

Table of Contents

1. Experimental Procedures S3

2. Preparation of model soot S3

3. Figure S1: Schematic diagram of the experimental set up S4

4. Preparation of $soot_{BaP}$ and $soot_{BaP+H_2SO_4}$ sample S4

5. Ozonation experiment S4

6. BaP Analysis S6

7. Results and Discussion S7

8. BaP ozonation kinetics on $soot_{BaP}$ samples S7

9. Figure S2: Ozonation kinetics of different BaP surface loads on soot S8

10. BaP ozonation kinetics in $soot_{BaP}$ and $soot_{BaP+H_2SO_4}$ samples S8

11. Figure S3: BaP ozonation kinetics in $soot_{BaP}$ and $soot_{BaP+H_2SO_4}$ samples S9

12. The accessibility of BaP in $soot_{BaP}$ and $soot_{BaP+H_2SO_4}$samples to gaseous O_3 S9

13. Figure S4: Prolonged exposure of soot adsorbed BaP to gaseous ozone S10

Experimental Procedures

Preparation of model soot. Figure S1a provides a simple set up for the collection of soot particles by burning kerosene. The detachable neck (A) of a simple wick lamp was attached to the mouth of a 25 mL borosilicate measuring cylinder (B) containing kerosene which is normally consumed for household use. The open end of a borosilicate glass tube (C, length 15 cm, diameter 2.5cm) was covered by Teflon sheet (thickness 2 mm) and fastened tight with a nylon cable tie. The lamp neck was inserted into the tube through a hole made at the center of the Teflon sheet. The cotton wick was pre-cleaned with dichloromethane (DCM, Emplura grade, Merck) in a Soxhlet apparatus for 4 hours and dried. The top opening was connected to the right-angled collection tube introduced inside a Buchner flask (D) filled with 200 mL n-hexane. The lamp was first allowed to burn for 15 minutes then its neck was carefully inserted inside the glass tube. To avoid background contamination, ultra pure air (99.999%, Bengal Gases, India) was introduced inside the glass tube through the side tube. The flowing air allowed the wick to burn and simultaneously acted as the carrier of the particulates into *n*-hexane. The length of the wick and the air flow rate (0.3 L min^{-1}) have been adjusted for a stable and medium flame for maximum soot collection and to avoid smothering of the flame with excessive smoke. The collection was continued until all the fuel was consumed (~ 6 hours). After collection, the *n*-hexane with the particulates was transferred into 50 mL Tarsoncentrifuge tubes and centrifuged with a Sorvall RC 6 Plus fixed rotor centrifuge for 30 minutes at the speed of 10000 rpm and $4^{O}C$. The n-hexane solution was decanted when black colored solid soot particles remained settled at the bottom. 40 mL DCM was then added to each tube and sonicated for 15 minutes at $18^{O}C$ followed by centrifugation. This step was repeated two more times.Finally, the particles were heated for 5 hrs inside a furnace at 400 ^{O}C to ensure maximum removal of the organic impurities. These final particles were termed as cleaned soot in this paper and used as substrate for heterogeneous BaP ozonation study. Approximately 1 g of carbon particle was generated by burning 10 mL kerosene.

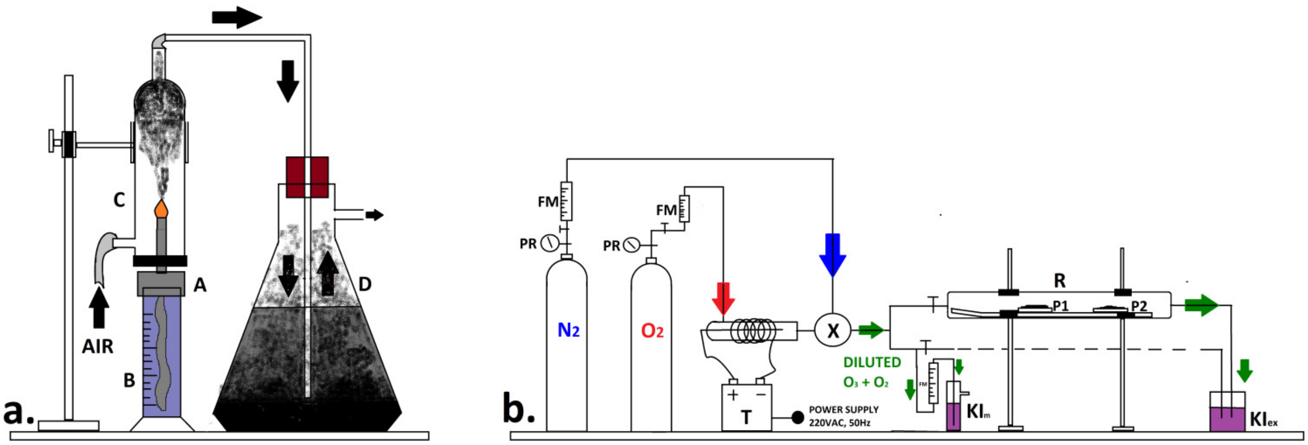

Figure S1: Schematic diagram of the experimental set up; (a) kerosene soot collection set up; (b) set up for BaP ozonation in $soot_{BaP}$ and $soot_{BaP+H_2SO_4}$ samples. (descriptions are given in the text)

Preparation of $soot_{BaP}$ and $soot_{BaP+H_2SO_4}$ sample. The cleaned soot particles were coated with BaP (≥ 96%, Sigma) by soaking the particles into a series of concentrations of BaP in DCM followed by blowing off DCM under gentle flow of N_2 gas. In this paper we described the BaP coated cleaned soot samples as$soot_{BaP}$. H_2SO_4 coated soot samples were prepared in the following way: 5 μL of ~ 98% H_2SO_4 (Emparta grade ACS, Merck, India; density = 1.84 gmL^{-1}) was pipetted into ~ 10 mL deionized water in a 25 mL glass beaker. Each time 10g of $soot_{BaP}$ was soaked into this solution. The water was completely dried off by heating the beaker at 180 ^{O}C. The resulting soot particles are coated with approximately monolayer coverage (estimated soot surface H_2SO_4 concentration was ~ 9×10^{-6} moles g^{-1}). In this paper we have described this $soot_{BaP}$ samples externally coated with H_2SO_4 as $soot_{BaP+H_2SO_4}$.

Ozonation experiment. Another experimental set up was developed to study the BaP ozonation kinetics. Figure S1b illustrates the schematic diagram of the experimental set up. The ozonation was carried out inside a quartz glass reactor (R, length: 30 cm; internal diameter: 3.5cm) with one end open able with glass socket. About 0.5 g of each sample ($soot_{BaP}$ and $soot_{BaP+H_2SO_4}$) was placed on rectangular Teflon plates (P1 and P2; length: 7cm, width: 2.5 cm, thickness: 0.2 cm; sample thickness: ~ 0.3 cm) attached on

a flat glass strip (length: 20cm, width: 2.5 cm). The glass strip (with P1 and P2) was inserted inside the reactor and the glass socket was closed. After ozonation, the glass strip was taken out to collect the exposed samples.

Ozone was produced by flowing ultra-high purity grade O_2 gas (99.999%, Bengal Gases, India) through non-thermal plasma generated inside a homemade dielectric barrier discharge (DBD) reactor. The reactor consists of a pyrex tube (length: 20 cm, diameter: 1.5cm and thickness: 0.2 cm) with one end sealed with glass and the other end having attached with a glass outlet tube (diameter: 3mm). The body of the pyrex tube was coaxially spiraled with a copper wire (length: 120 cm, diameter: 0.3 mm) which was used as cathode. Another copper wire (length: 15 cm, diameter: 0.3 mm) which was inserted through the other glass sealed end of the pyrex tube, was used as anode. The plasma was generated by using a high voltage current limiting transformer typically used in neon signs. An alternating current of 15 kV at 50Hz was used between the two electrodes separated by a dielectric medium to generate cold plasma. The O_2 gas pressure from the cylinder was regulated at 2.0 bars with a pressure regulator (PR, Apollo Engineering, India), the flow rates were controlled by glass stopcock and measured by a flow meter (FM, SS Flow, India) followed by introduced into the DBD reactor through a glass inlet tube as shown in Figure S1b. The ozone concentrations were varied by diluting the O_3+O_2 mix with regulated flow of N_2 gas inside a glass bulb (X, diameter: 3 cm). The flow rate of diluted O_3+O_2 mix exiting from the glass bulb was maintained at 0.3 L min^{-1} by adjusting the flow rates of O_2 and N_2 gas. The diluted O_3+O_2 mix flow was splited into two parts ─ one part was allowed to enter the reactor (R) and the exhaust was directed into potassium iodide solution (KI_{ex}). The ozone concentrations were determined iodometrically from a flow controlled fraction of the other part[1,2] and the rest was exhausted into potassium iodide solution (KI_{ex}). A flow-controlled fraction of the (O_3+O_2) mix was directed into a gas bubbler containing buffered solution of 2% potassium iodide (KI_m) for an exact time period[1]. Equivalent moles of triiodide ions (I_3^-) are formed as that of ozone at excess iodide concentration, therefore I_3^- concentrations were determined spectrophotometrically (absorbance at 351nm) from the absorbance vs. concentration calibration curve, which was prepared by using standard I_3^- solutions.[2] The O_3concentrations were measured before and

after the experiments and were always corrected for KI oxidation by molecular oxygen. The O_3 concentrations inside the reactor (R) were in the range of (0.2 – 9) × 10^{15}molecules cm^{-3}.

BaP Analysis. After exposure to ozone, each sample was sonicated with 5 mL DCM for 30 mintutes followed by centrifugation at 10000 rpm for 30 minutes in a 10 mL Tarsoncentrifuge tube with a Sorvall RC 6 Plus fixed rotor centrifuge. The solution was decanted and stored in a 40 mL glass vial. The step was repeated four more times. Each of the 25 mL extract was then first solvent exchanged to 5 mL acetonitrile followed by concentrated to approximately 1 mL by gentle flow of nitrogen. Every sample was then spiked with internal standard and stored in a 2 mL amber glass vial at 4 ^{O}C until the analysis.
The extracts were analyzed with a high performance liquid chromatography (HPLC, Shimadzu Prominance) with fluorescence detector (Shimadzu RF 10AXL). 2 µL extract was injected into the system equipped with a C-18 reversed-phase chromatographic column (Supelcosil™ LC-PAH, 15 cm × 4.6 mm, 5 µm) and the analysis was carried out using an isocratic elution of acetonitrile-water (97:3 v/v, Gradient grade LiChrosolv® Merck, India and 18 MΩ water, respectively) at a constant flow rate of 1 mL min^{-1}. The BaP signal was monitored at excitation/detection wavelength of 297/404 nm and chromatographic peak integrations were performed with LCsolution software (Shimadzu). Along with each measurement, a set of BaP standard solutions (0.2 – 100 ppm) was run and BaP was quantified from the calibration curve. Around 83% recovery was observed for solvent blank experiments and subsequent corrections were performed on the HPLC peak areas prior to the calculations of the concentrations from the calibration curve. The method detection limit of BaP quantification was 0.18 ppm.

Results and Discussion

BaP ozonation kinetics on $\mathbf{soot_{BaP}}$ samples. Figure S2 illustrates the BaP ozontion kinetics in $soot_{BaP}$ samples with a series of BaP surface loads at ~ 8ppm ozone concentration. The kinetics was studied by quantifying BaP degradation with increasing ozone exposure time. The reaction was observed to follow first order kinetics as described by the linear plots (Figure S2). The observed pseudo-first order rate constants (k_{obs}^{I}) were evaluated from the slopes of linear least-squares-fits for each BaP surface load and the values are shown in the figure. Additionally, control experiments were carried out to evaluate the BaP loss through evaporation and oxidation by molecular oxygen which may interfere in BaP ozonation kinetics. The loss by evaporation was evaluated by flowing nitrogen gas (flow rate: 0.3 $Lmin^{-1}$) through the reactor keeping other experimental conditions similar. Insignificant loss of BaP (< 1%) from the $soot_{BaP}$ samples was quantified after 3 minutes of exposure to N_2 gas for both small (c_{Bap}^{0} = 3.12 x 10^{-6} moles g^{-1}) and large (c_{Bap}^{0} = 8.9 x 10^{-5} moles g^{-1}) BaP surface loads. Similarly in order to evaluate the BaP loss due to oxidation by molecular oxygen, only O_2 gas was flown (flow rate: 0.3 $Lmin^{-1}$) through the reactor. Thus including the evaporation effect, the BaP loss due to oxidation by molecular O_2 is < 5% after 3 minutes for both small and large BaP surface loads. Thereafter, all the experimental data are corrected by considering the loss of BaP as evaluated in the control experiments.

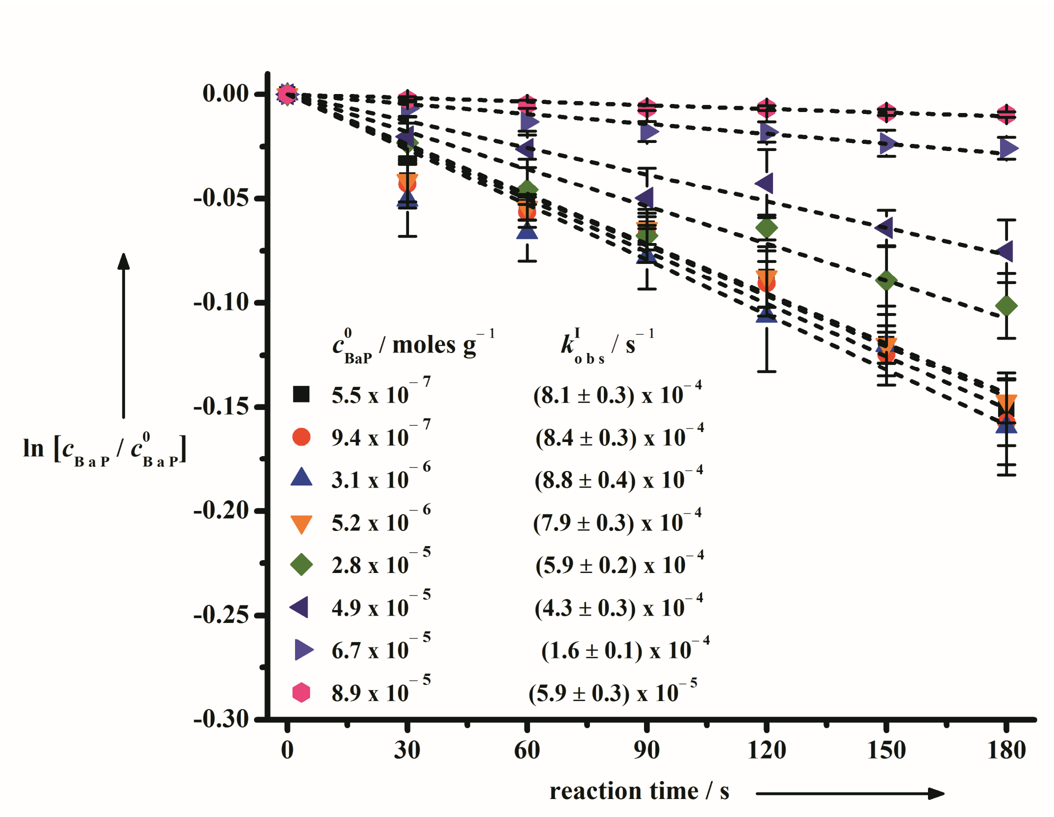

Figure S2: Determination of the observed pseudofirst order ozonation (c_{O_3}= 2 x 10^{14}molecules cm^{-3}) rate constants (k^{I}_{obs}) of BaP on cleaned soot by plotting $\ln[c_{BaP}/c^{0}_{Bap}]$ *vs* reaction time using the least-squares method for a series of initial BaP surface loads, c^{0}_{Bap} (molesg^{-1}); c_{BaP} (molesg^{-1}) are the BaP concentrations during ozonation; error bars represent the standard deviation

BaP ozonation kinetics in soot$_{BaP}$ and soot$_{BaP+H_2SO_4}$ samples. The ozonation kinetics of sub-monolayer concentration of BaP (c^{0}_{Bap} = 3.12 x 10^{-6}moles g^{-1}, corresponding to ~ 0.4 times monolayer) in soot$_{BaP}$ and soot$_{BaP+H_2SO_4}$ samples exposed to a range of ozone concentrations (c_{O_3}= 8 — 360 ppm) are illustrated in Figure S3 (a–b) respectively. The average values of c^{0}_{Bap} and corresponding evaluated k^{I}_{obs} values are shown in the figure.

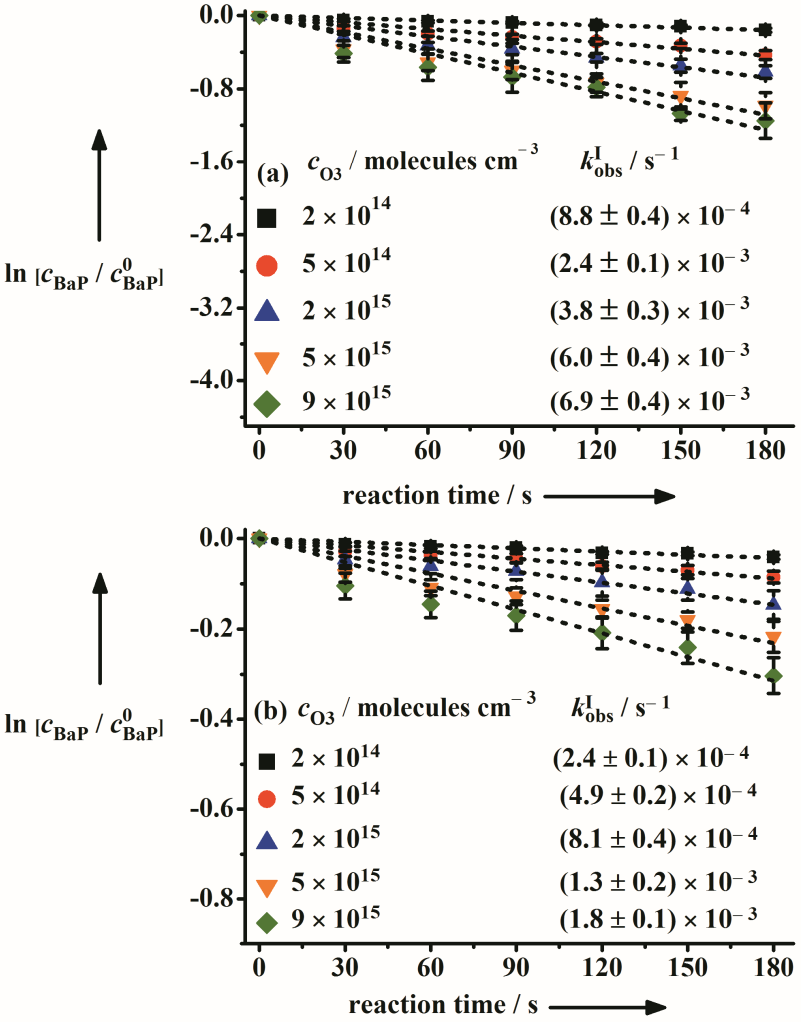

Figure S3: Determination of the observed pseudo first order ozonation rate constants (k^{I}_{obs}) of BaP (c^0_{Bap} = 3.12x10^{-6}moles g^{-1}) in soot$_{BaP}$ and soot$_{BaP+H_2SO_4}$ samples by plotting $\ln[c_{BaP}/c^0_{Bap}]$ *vs* reaction time using the least-squares method for a series of initial BaP surface loads; c^0_{Bap} (molesg^{-1}); c_{BaP} (moles g^{-1}) are the BaP concentrations during ozonation; error bars represent the standard deviation

The accessibility of BaP in soot$_{BaP}$ and soot$_{BaP+H_2SO_4}$ samples to gaseous O$_3$. We also tested the accessibility of BaP (c^0_{Bap} = 3.12 x 10^{-6}moles g^{-1}, corresponding to ~ 0.4 times monolayer) molecules in soot$_{BaP}$and soot$_{BaP+H_2SO_4}$ samples to gaseous ozone (c_{O_3}= 2 x 10^{14}molecules cm^{-3}) by prolonging the ozone exposure time (Figure S4). In order to evaluate the BaP loss from soot$_{BaP}$ and soot$_{BaP+H_2SO_4}$

samples by evaporation and oxidation due to molecular oxygen, we have also performed control experiments (vide supra). Less than 3% and < 1% BaP loss were observed due to evaporation after 3 hours from $soot_{BaP}$ and $soot_{BaP+H_2SO_4}$ samples respectively, whereas including the loss due to evaporation, < 12% and < 5 % BaP loss was evaluated due to oxidation by O_2 gas after 3 hours from $soot_{BaP}$ and $soot_{BaP+H_2SO_4}$ samples respectively. The control experiments were performed on $soot_{BaP}$ and $soot_{BaP+H_2SO_4}$ samples with submonolayer BaP surface loads. The experimental data were subsequently corrected with the results from the control experiments. Almost 85% of BaP in the $soot_{BaP}$ samples degraded in 3 hours, indicating that most of the BaP molecules are accessible to gaseous O_3. On the other hand, < 30% of BaP degradation was observed in $soot_{BaP+H_2SO_4}$ samples indicating significant influence of the H_2SO_4 coating on the accessibility of surface adsorbed BaP to gaseous O_3.

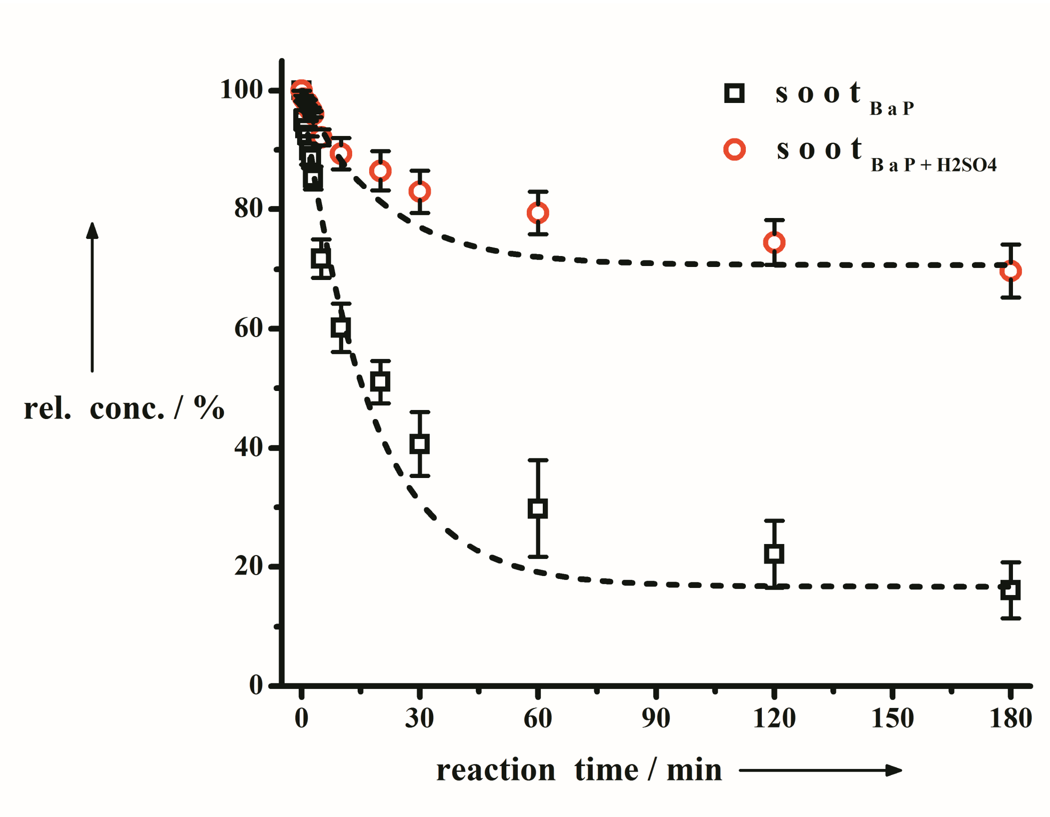

Figure S4: Relative concentrations of BaP (c^0_{BaP} = 3.12x10^{-6}moles g^{-1}) during ozonation (c_{O_3}= 2 x 10^{14}molecules cm^{-3}) on $soot_{BaP}$ and $soot_{BaP+H_2SO_4}$ samples inside the reactor at room temperature and pressure; error bars represent the standard deviation.

References

1 Flamm, D. L. Analysis of ozone at low concentrations with boric acid buffered potassium iodide. *Environmental Science & Technology* 11, 978-983, (1977).

2 Ray, D., Kurková, R., Hovorková, I. & Klán, P. Determination of the Specific Surface Area of Snow Using Ozonation of 1,1-Diphenylethylene. *Environmental Science & Technology* 45, 10061-10067, (2011).
